# Preemptive Propofol Administration in Spinal Cord Injury: Effects on Pain‐Induced Hypertension, Neuroinflammation, and Functional Recovery in Rats

**DOI:** 10.1002/kjm2.70011

**Published:** 2025-04-01

**Authors:** Qun Cheng, Xiang‐Yu Fang, Rong‐En Qiu

**Affiliations:** ^1^ Anesthesiology Department The Quzhou Affiliated Hospital of Wenzhou Medical University, Quzhou People's Hospital Quzhou China

**Keywords:** anesthesia, general, models, animal, propofol, recovery of function, spinal cord injuries

## Abstract

Spinal cord injury (SCI) triggers secondary damage, including pain‐induced hypertension, inflammation, and hemorrhage, impairing recovery. This study evaluated the efficacy of general anesthesia with preemptive propofol administration in mitigating secondary damage in SCI rats. SCI was induced in rats using a contusion model. Propofol (100 mg/kg) was administered intraperitoneally either 30 min before (preemptive) or 30 min after intermittent tail shock. Systolic blood pressure (SBP), body weight, food intake, inflammatory markers (interleukin‐1 beta [IL‐1β], interleukin‐6 [IL‐6]), hemorrhage markers, and serum levels of SCI biomarkers (glial fibrillary acidic protein [GFAP], myelin basic protein [MBP]) were measured. Functional recovery was assessed over 28 days using the Basso, Beattie, and Bresnahan (BBB) scale, horizontal ladder test, and rotarod test. Preemptive propofol administration effectively mitigated pain‐induced hypertension, enhanced body weight and food intake, and reduced inflammatory markers to levels comparable to unstimulated SCI rats. In contrast, propofol administered post‐stimulation was less effective. Preemptive administration significantly decreased GFAP levels and preserved MBP levels. Importantly, preemptive intervention reduced levels of hemoglobin and alpha hemoglobin, while post‐stimulation intervention showed no significant effect on hemorrhage. Behavioral assessments demonstrated improved locomotor recovery, motor coordination, and balance in preemptively treated rats compared to delayed or no intervention. Preemptive administration of propofol effectively reduces pain‐induced hypertension, inflammation, and gliosis while preserving myelin integrity and enhancing functional recovery in SCI rats. This intervention demonstrates significantly greater efficacy compared to delayed administration, underscoring the critical importance of timely treatment in mitigating secondary damage and improving outcomes after SCI.

## 1 Introduction

Spinal cord injury (SCI) is a severe and often life‐altering condition that leads to a complex cascade of primary and secondary injury mechanisms, resulting in significant neurological deficits and functional impairments [1]. The primary injury phase, which occurs within minutes to hours, results from immediate mechanical forces delivered to the spinal cord, causing direct structural damage to neuronal and vascular tissues, leading to acute cell dysfunction and death [2]. The secondary injury phase, beginning shortly after the initial trauma, continues for days to weeks or even months and is characterized by a cascade of biochemical events, including ischemia, oxidative stress, inflammation, excitotoxicity, and neuronal apoptosis, that exacerbate the initial damage [3]. These secondary processes contribute to further tissue destruction and functional impairments, underscoring the critical need for timely intervention, especially within the first 24–48 h post‐injury, when these processes are at their peak and intervention can effectively prevent the progression of secondary damage. Despite advances in understanding SCI pathophysiology, a significant gap remains in effective therapies to mitigate secondary processes and promote recovery, emphasizing the need for novel neuroprotective agents targeting these underlying mechanisms to improve SCI prognosis.

One of the key contributors to secondary damage in SCI is pain‐induced hypertension, which arises from autonomic dysregulation and persistent nociceptive input [4]. Autonomic dysregulation, particularly an overactive sympathetic nervous system, triggers a hypertensive response that exacerbates blood pressure fluctuations and significantly contributes to the breakdown of the blood‐spinal cord barrier (BSCB) [5, 6]. Pain input, particularly caudal to a spinal cord contusion injury, further worsens tissue loss by disrupting the BSCB, allowing blood components such as hemoglobin to infiltrate the injury site, thereby increasing hemorrhage [7]. This process amplifies oxidative stress and the release of pro‐inflammatory cytokines, triggering a cascade of pathological events that lead to apoptosis, further BSCB disruption, cerebral edema, and hemorrhagic transformation [8]. Additionally, neuroinflammation, characterized by the activation of glial cells such as microglia and astrocytes, promotes the release of pro‐inflammatory cytokines and chemokines, exacerbating neuronal injury and limiting the regenerative capacity of the spinal cord [9]. Collectively, these processes disrupt both motor and sensory pathways, leading to long‐term motor coordination deficits and sensory impairments that are common in SCI patients [10]. These findings highlight the urgent need for early intervention, particularly within the first 24–48 h post‐injury, to control pain‐induced hypertension and prevent further vascular and neuronal damage, thereby improving long‐term functional outcomes.

General anesthesia has emerged as a potential strategy to mitigate secondary damage in SCI due to its ability to modulate nociceptive pathways and reduce systemic stress responses [4, 11]. Propofol, a widely used intravenous anesthetic, has gained attention for its neuroprotective properties beyond its primary sedative effects [12]. Propofol was reported to exert anti‐inflammatory, antioxidant, and anti‐apoptotic effects, thus ameliorating the SCI process [13]. These properties make it a promising candidate for mitigating secondary damage in SCI. The timing of anesthetic administration is crucial in determining the extent of protective effects, with preemptive treatment proving to be significantly more beneficial than delayed administration. For example, preemptive anesthesia (whether with pentobarbital or isoflurane) played a protective role in attenuating pain‐induced hemorrhage, controlling inflammatory responses, and modulating systemic physiological responses (e.g., blood pressure) following SCI. Delayed anesthesia was ineffective in preventing hemorrhage and inflammatory cytokine release, reinforcing the notion that early intervention is critical to preventing secondary damage after SCI [4].

In the present study, we hypothesize that preemptive administration of propofol under general anesthesia would provide superior neuroprotection by mitigating pain‐induced hypertension, inflammation, hemorrhage, and gliosis, while preserving myelin integrity and enhancing recovery in SCI. The objectives of this study are to evaluate the therapeutic efficacy of preemptive versus delayed propofol administration in reducing secondary damage and improving functional outcomes in SCI rats. By focusing on propofol's role in general anesthesia, this study seeks to establish its potential as a timely and effective intervention for secondary injury mitigation in SCI.

## 2 Materials and Methods

### 2.1 Experimental Animals

Adult male Sprague–Dawley rats were used in this study. Before the experiments, the rats underwent a 7‐day acclimatization period to minimize handling stress. During this time, the animals were housed under a 12‐h light/dark cycle (humidity: 40%–60%, temperature: 22°C) with free access to standard laboratory chow (Teklad 8460 diet, energy density: 12.6 kJ/g, 3.0 kcal/g) and water. All experiments were conducted in accordance with the National Institutes of Health (NIH) guidelines for the care and use of laboratory animals [14] and were approved by the Ethics Committee of our institution.

### 2.2 Spinal Cord Injury (SCI) Modeling

Rats were anesthetized with 5% isoflurane in 95% oxygen in a Plexiglass chamber for induction. During surgery, anesthesia was maintained with 2.5% isoflurane in oxygen via an inhalation mask connected to a vaporizer (flow rate: 0.4 L/min; Medical Supplies and Services). Adequate anesthesia depth was confirmed by the absence of the toe pinch reflex. After induction, the dorsal fur was shaved, and the rat was secured on a surgical table with body temperature maintained using a heating pad and monitored with a rectal thermometer. A spinal cord contusion model was established at the T10 vertebral level using the Multicenter Animal Spinal Cord Injury Study Impactor (MASCIS, W.M. Keck Center for Collaborative Neuroscience, Piscataway, NJ, USA). A 3 cm longitudinal incision was made along the midline of the back, and the muscles and soft tissues surrounding the spinous processes of T11 –T12 vertebrae were dissected using microsurgical instruments to expose the spinal cord. The vertebrae were stabilized on both sides using the MASCIS clamps. A 10 g weight was released from a height of 12.5 mm to induce spinal cord contusion [4]. Post‐surgery, the incision was closed with Michel stainless steel clips, and the rats received a subcutaneous injection of 3 mL of sterile saline to prevent dehydration. Penicillin (100,000 units/kg) was administered subcutaneously to prevent infection. Rats were housed individually in temperature‐controlled cages with free access to food and water. Health status was monitored during the 24‐h recovery period.

### 2.3 Experimental Design and Sample Size Calculation

**FIGURE 1:**
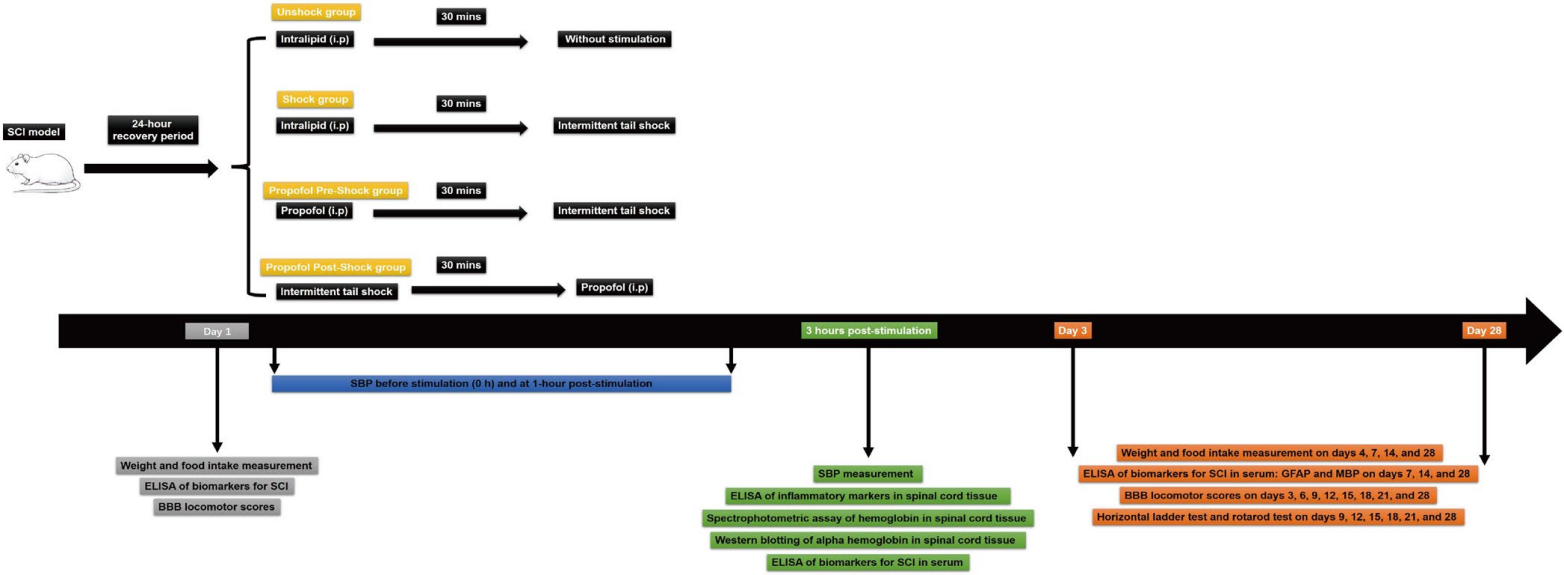
Experimental design, group assignments, and study timeline. Illustration of the experimental design, including the group assignments (Unshock, Shock, Propofol Pre‐Shock, and Propofol Post‐Shock) and the study timeline for measuring outcome variables, such as systolic blood pressure (SBP), inflammatory markers, hemorrhage markers, spinal cord injury (SCI) biomarkers, and functional recovery assessments.

SCI rats were randomly divided into four groups (Figure 1): (1) Unshock group: received an equal volume of intralipid solution (10% fat emulsion) without pain stimulation; (2) Shock group: received intralipid solution 30 min before pain stimulation; (3) Propofol Pre‐Shock group: received 100 mg/kg propofol [15, 16] (Propofol, Sigma‐Aldrich, Saint Louis, MO) dissolved in 10% fat emulsion 30 min before pain stimulation; (4) Propofol Post‐Shock group: received 100 mg/kg propofol immediately after pain stimulation. Rats were randomly assigned to the four groups using a computer‐generated randomization schedule to ensure unbiased allocation. Sample size per group was calculated using the formula *N* = (DF/*κ*) + 1, where *N* is the sample size per group, DF is the degrees of freedom, and *κ* is the number of groups [17]. In this formula, the DF typically ranges from 10 to 20, balancing statistical power and practical constraints such as resource availability [18]. To ensure statistical power, two separate experiments were conducted, including biochemical analysis and behavioral analysis, with six rats per group. A total of 48 rats (24 for each experiment) were included to maintain methodological consistency and robust statistical comparisons. Body weight and food intake were monitored throughout the study. All evaluations were performed by blinded assessors to ensure objectivity and minimize bias.

### 2.4 Intermittent Tail Shock

To induce secondary injury, uncontrollable electrical stimulation was applied to the tail 24 h after SCI. The animals were restrained in opaque Plexiglas tubes, which were placed in a soundproof chamber. The tail was secured to an electrode approximately 4 cm from the tip, and conductive gel was applied to ensure good contact between the electrode and the tail. The electrical stimulation parameters were as follows: a 660 V AC power source provided a 60 Hz alternating current signal. Each stimulus lasted 100 ms, with inter‐stimulus intervals randomly distributed between 0.2 and 3.8 s. The stimulation was delivered continuously for 6 min. Control animals were restrained in the same manner but did not receive electrical stimulation. Blood pressure was measured using the CODA High Throughput Noninvasive Blood Pressure System and data acquisition software (Kent Scientific). Blood pressure values were recorded at three time points: before stimulation (0 h), 1 h after stimulation, and 3 h after stimulation.

### 2.5 Sample Collection

Three hours after intermittent tail shock, rats were euthanized via intraperitoneal injection of sodium pentobarbital (800 mg/kg; Euthanyl, 240 mg/mL, Bimeda‐MTC Animal Health, Cambridge, Ontario, Canada) [19]. The spinal cord tissue, including the injury site and 0.5 cm segments both rostral and caudal to the lesion (total length: 1 cm), was carefully harvested. Collected tissue was immediately frozen in liquid nitrogen and stored at −80°C for further analysis. Blood samples were drawn from the tail vein at 3 h after intermittent tail shock, as well as on days 1, 7, 14, and 28 post‐SCI. Samples were transferred into sterile tubes containing EDTA as an anticoagulant. The tubes were centrifuged at 3000 × g for 10 min at 4°C to separate serum, which was aliquoted and stored at −80°C for subsequent biochemical analyses.

### 2.6 Assessment of Hemorrhage Markers (Hemoglobin and Alpha Hemoglobin)

Hemoglobin content in spinal cord tissue was quantified using the QuantiChrom Hemoglobin Assay Kit (DIHB‐250, BioAssay Systems). Tissue was homogenized in ice‐cold 0.1 mol/L PBS, centrifuged at 13,000 × g for 30 min at 4°C, and supernatants were collected. Samples were loaded into a 96‐well plate alongside blanks and standards, followed by the addition of 200 μL of hemoglobin reagent. After 5 min of incubation at room temperature, optical density was measured at 400 nm using a spectrophotometer (Ultrospec 2000, Pharmacia Biotech, UK). For Western blot analysis, spinal cord lysates were prepared in RIPA buffer with protease inhibitors, and protein concentration was determined using a BCA assay. Equal amounts of protein (20–30 μg) were separated on 10% SDS‐PAGE gels, transferred to PVDF membranes, and blocked with 5% nonfat dry milk in TBST. Membranes were incubated overnight at 4°C with anti‐hemoglobin subunit alpha antibody (1:2000, Abcam) and subsequently with HRP‐conjugated goat anti‐rabbit secondary antibody (1:2000) for 1 h. β‐actin (1:5000, Abcam) was used as a loading control. Protein bands were visualized using enhanced chemiluminescence (ECL).

### 2.7 Enzyme‐Linked Immunosorbent Assay (ELISA)

Serum levels of glial fibrillary acidic protein (GFAP, MBS700870, inter−/intra‐assay variations: < 8%/< 10%; MyBioSource, CA, USA) and myelin basic protein (MBP, MBS007623, inter−/intra‐assay variations: < 15%/< 15%; MyBioSource) were measured using ELISA. Additionally, rat‐specific ELISA kits were used to quantify inflammatory cytokines in spinal cord tissue, including interleukin‐1 beta (IL‐1β, ERIL1BX10), interleukin‐6 (IL‐6, ERA32RB), and tumor necrosis factor‐alpha (TNF‐α, ERA57RB), all with inter−/intra‐assay variations of < 12%/< 10% (Thermo Fisher Scientific, USA). Spinal cord lysates were prepared by homogenizing tissue in radioimmunoprecipitation assay (RIPA) buffer supplemented with 1% Triton X‐100 and a protease inhibitor cocktail. The homogenates were centrifuged at 10,000 × g for 5 min at 4°C, and the supernatants were aliquoted and stored at −80°C for further analysis.

### 2.8 Behavioral Examinations

Locomotor function was assessed using the Basso, Beattie, and Bresnahan (BBB) Locomotor Rating Scale, Horizontal Ladder Test, and Rotarod Test after a lower thoracic spinal contusion (day 1). The BBB scale, ranging from 0 (fully paralyzed) to 21 (complete recovery), was conducted in an open field for 4 min [20]. BBB scores were recorded daily on days 1, 3, 6, 9, 12, 15, 18, 21, and 28. In the Horizontal Ladder Test, hind‐limb placement accuracy was analyzed as rats traversed a 1‐m ladder with 1‐cm spaced rungs, with performance scored on a 7‐point scale (0–6) [21]. The Rotarod Test (IITC Life Science, WPI, UK) measured the time and speed at which rats fell from an accelerating rod (0–30 rpm over 120 s), assessing balance and motor endurance [22]. Both tests were conducted on days 9, 12, 15, 18, 21, and 28 post‐injury.

### 2.9 Statistical Methods

All statistical analyses were conducted using GraphPad Prism (GraphPad Software, San Diego, CA, USA). Results are expressed as mean ± standard deviation (SD). Inter‐group comparisons were performed using one‐way or two‐way analysis of variance (ANOVA), followed by Tukey's post hoc test for multiple comparisons. Inflammatory markers were compared across groups using one‐way ANOVA. Two‐way ANOVA was employed to analyze data involving multiple factors, such as treatment and time, across behavioral assessments and other outcomes. A significance threshold of *p <* 0.05 was applied for all analyses.

## 3 Results

### 3.1 Preemptive Propofol Mitigates Pain‐Induced Hypertension in SCI Rats

**FIGURE 2:**
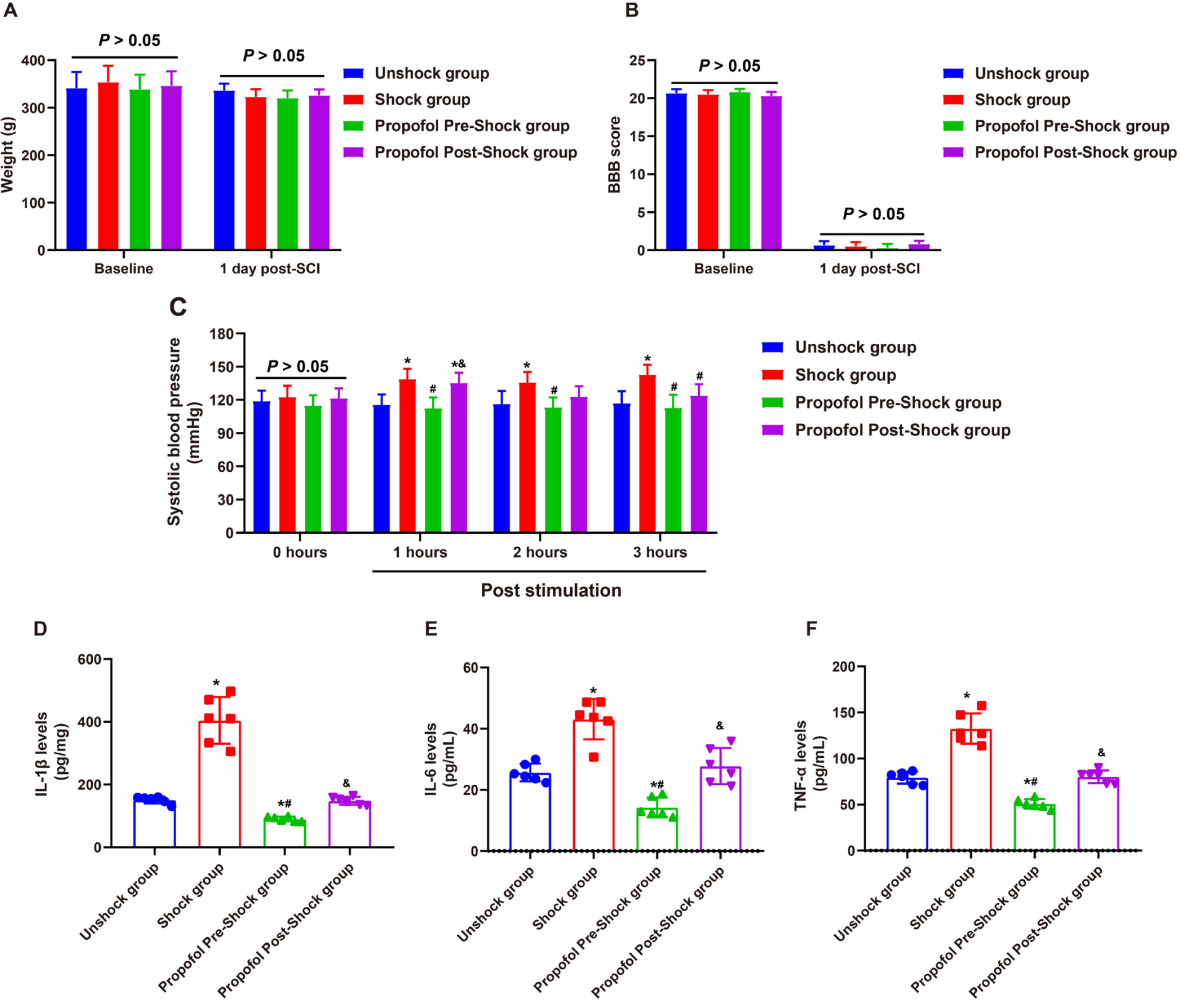
Effects of general anesthesia with preemptive propofol on pain‐induced hypertension and inflammation in SCI rats. (A) Body weight of rats at baseline and 1‐day post‐ spinal cord injury (SCI); (B) Basso, Beattie, and Bresnahan (BBB) locomotor rating scale scores assessed at baseline and 1‐day post‐SCI; (C) Systolic blood pressure (SBP) measurements at baseline (0 h), 1, and 3 h post‐stimulation. (D–F) Levels of inflammatory markers interleukin‐1 beta (IL‐1β) (D), interleukin‐6 (IL‐6) (E), and tumor necrosis factor‐alpha (TNF‐α) (F) in spinal cord tissue measured 3 h post‐stimulation. Data are presented as mean ± SD. *p < 0.05 versus. Unshock group; #*p <* 0.05 versus Shock group; &*p <* 0.05 versus Propofol Pre‐Shock group. *N* = 6.

There were no significant differences in body weight or BBB scores between the four groups at baseline or day 1 post‐SCI (Figure 2A,B). At 0 h, prior to pain stimulation (intermittent tail shock), SBP levels were comparable across all groups (*p >* 0.05). At 1 h post‐stimulation, the Shock group exhibited a significant hypertensive response compared to the Unshock group (*p <* 0.05). Pre‐treatment with propofol 30 min before stimulation effectively mitigated this response, maintaining SBP levels comparable to the Unshock group (*p >* 0.05). In contrast, post‐stimulation propofol administration resulted in a transient SBP increase (*p <* 0.05), which normalized by 2–3 h post‐stimulation (*p >* 0.05). At 2 and 3 h, SBP remained elevated in the Shock group compared to the Unshock group (*p <* 0.05), while pre‐treated rats exhibited normotensive levels, underscoring the greater efficacy of preemptive intervention (Figure 2C).

### 3.2 Preemptive Propofol Mitigates Pain‐Induced Inflammation in SCI Rats

The inflammatory response, assessed through IL‐1β, IL‐6, and TNF‐α levels in spinal cord tissue 3 h post‐stimulation, revealed significant increases in both markers following pain stimulation compared to unstimulated rats (*p <* 0.05). Preemptive propofol administration markedly attenuated these elevations, resulting in levels significantly lower than those in untreated stimulated rats (*p <* 0.05) and comparable to unstimulated rats (*p >* 0.05). Conversely, post‐stimulation propofol administration provided only partial suppression, with inflammatory marker levels significantly higher than those in pre‐treated rats (*p <* 0.05) and not significantly different from untreated stimulated rats (*p >* 0.05, Figure 2D–F). These findings collectively highlight the superior efficacy of preemptive propofol administration in mitigating pain‐induced hypertension and inflammation in SCI rats.

### 3.3 Preemptive Propofol Mitigated Pain‐Induced Hemorrhage in SCI Rats

**FIGURE 3:**
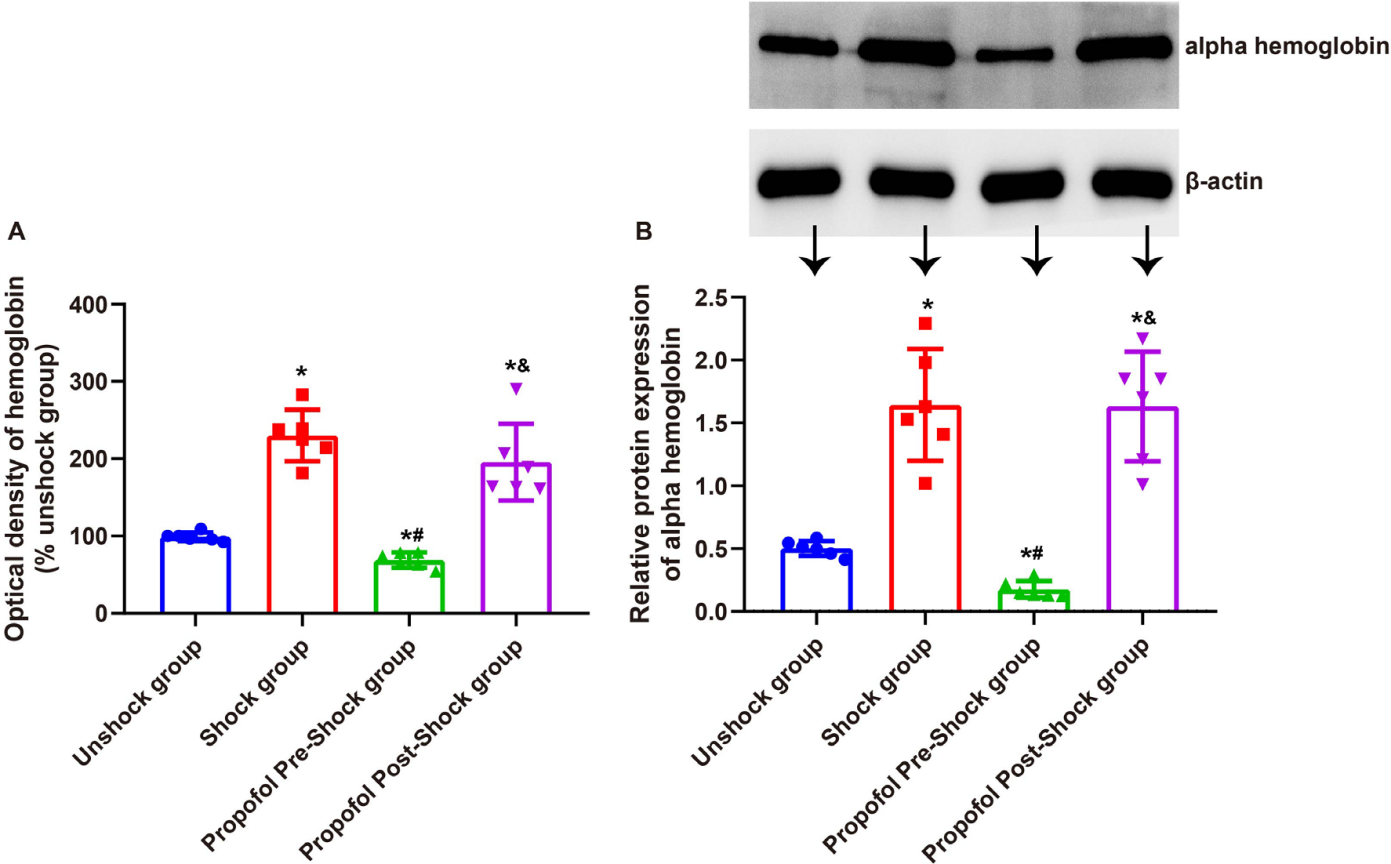
Effects of general anesthesia with preemptive propofol on hemorrhage markers in SCI rats. (A) Hemoglobin levels in spinal cord tissue measured 3 h post‐stimulation using a spectrophotometric assay. (B) Western blot analysis of alpha hemoglobin expression in spinal cord tissue 3 h post‐stimulation. Data are presented as mean ± SD. *p < 0.05 versus Unshock group; #*p <* 0.05 versus Shock group; &*p <* 0.05 versus Propofol Pre‐Shock group. *N* = 6.

In spinal cord tissue of SCI rats, analyzed 3 h after intermittent tail shock, spectrophotometric assay of hemoglobin (Figure 3A) and Western blotting of alpha hemoglobin (Figure 3B) revealed significant nociception‐induced hemorrhage. Both hemoglobin levels and alpha hemoglobin expression were markedly elevated compared to baseline conditions (*p <* 0.05). Preemptive administration of propofol prior to nociceptive stimulation effectively attenuated these changes, restoring measurements to levels comparable to baseline (*p >* 0.05) and significantly reducing hemorrhage relative to untreated conditions (*p <* 0.05). In contrast, post‐stimulation administration of propofol provided minimal protection, with hemoglobin levels and alpha hemoglobin expression remaining elevated and comparable to untreated rats (*p >* 0.05). These findings highlight the critical role of timely intervention in mitigating nociception‐induced hemorrhage in SCI rats.

### 3.4 Effect of Propofol Intervention Prior to Pain Stimulation on Spinal Cord Injury Markers in SCI Rats

**FIGURE 4:**
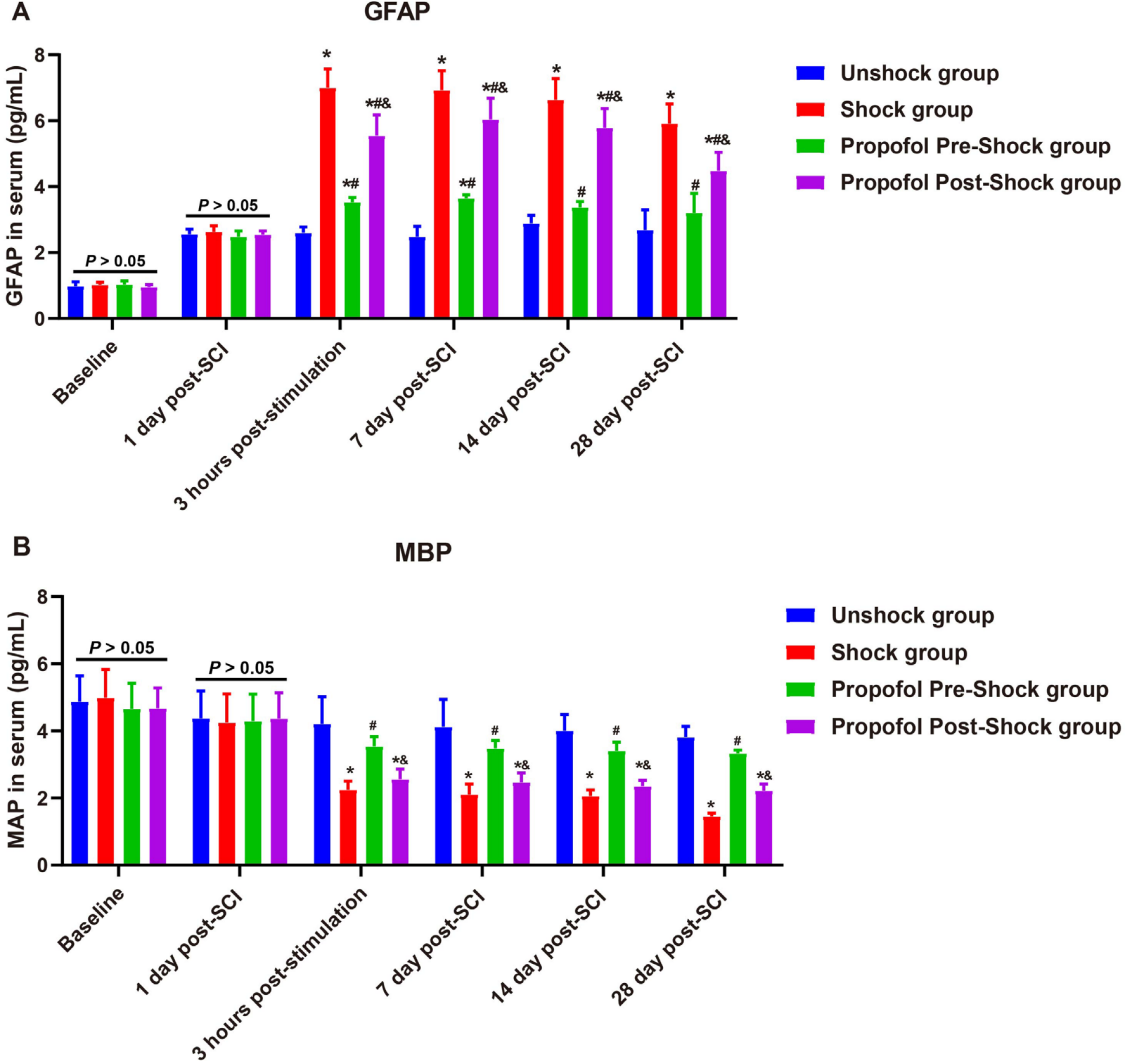
Effects of general anesthesia with preemptive propofol on serum biomarkers of spinal cord damage in SCI rats. (A) Serum levels of glial fibrillary acidic protein (GFAP) measured at baseline, 1‐day post‐SCI, 3 h post‐stimulation, and subsequent time points. (B) Serum levels of myelin basic protein (MBP) measured at the same time points. Data are presented as mean ± SD. *p < 0.05 versus Unshock group; #*p <* 0.05 versus Shock group; &*p <* 0.05 versus Propofol Pre‐Shock group. *N* = 6.

Serum GFAP and MBP levels in SCI rats showed no significant differences at baseline or 1 day post‐SCI, confirming similar starting conditions (all *p >* 0.05). From 3 h post‐stimulation onward, pain stimulation significantly elevated GFAP levels and reduced MBP levels (*p <* 0.05). Early propofol intervention prior to stimulation mitigated these changes, leading to lower GFAP levels and higher MBP levels compared to delayed or no intervention (Figure 4A,B). Over time, GFAP levels decreased across all groups but remained highest in those with delayed or no intervention, reflecting prolonged astrocytic activation. Similarly, MBP levels continued to decline by 7 and 14 days post‐SCI, with the greatest loss in the pain‐stimulated group without intervention. By 28 days, MBP levels partially recovered, but early propofol intervention consistently preserved myelin integrity better than delayed administration (*p <* 0.05). These findings highlight the protective effects of timely propofol administration in reducing gliosis and preserving myelin in SCI.

### 3.5 Propofol Intervention Prior to Pain Stimulation Enhances Body Weight and Food Intake in SCI Rats

**FIGURE 5:**
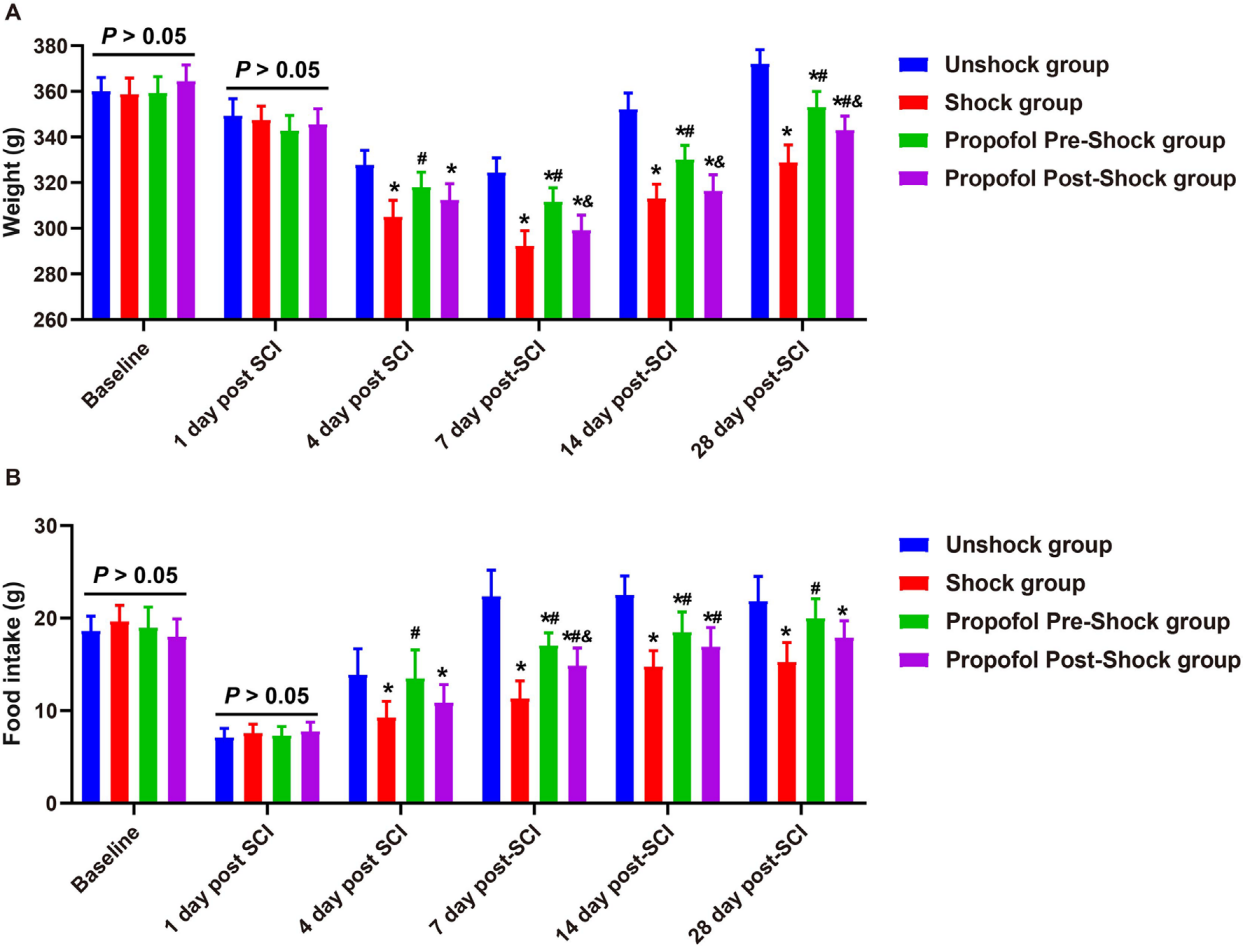
Effects of general anesthesia with preemptive propofol on body weight and food intake in SCI rats. (A) Body weight changes in SCI rats at baseline and 1‐, 4‐, 7‐, 14‐, and 28‐days post‐SCI; (B) Food intake changes in SCI rats at baseline and 1‐, 4‐, 7‐, 14‐, and 28‐days post‐SCI; Data are presented as mean ± SD. *p < 0.05 versus Unshock group; #*p <* 0.05 versus Shock group; &*p <* 0.05 versus Propofol Pre‐Shock group. *N* = 6.

Body weight and food intake were monitored at several time points. No significant differences in body weight or food intake were observed among the four groups at baseline or 1 day post‐SCI (all *p* > 0.05, Figure 5A,B). At 4 and 7 days post‐SCI, the Unshock group rats showed a reduction in body weight, which gradually recovered by 14 and 28 days post‐SCI. These changes in body weight were accompanied by corresponding alterations in food intake, suggesting that the initial weight loss following SCI was compensated for by an increase in food intake [23]. Pain stimulation led to a reduction in both body weight and food intake compared to the Unshock group. However, rats that received propofol prior to pain stimulation demonstrated significantly higher body weight and food intake compared to those treated with propofol after stimulation.

### 3.6 Propofol Intervention Prior to Pain Stimulation Enhances Functional Recovery in SCI Rats

**FIGURE 6:**
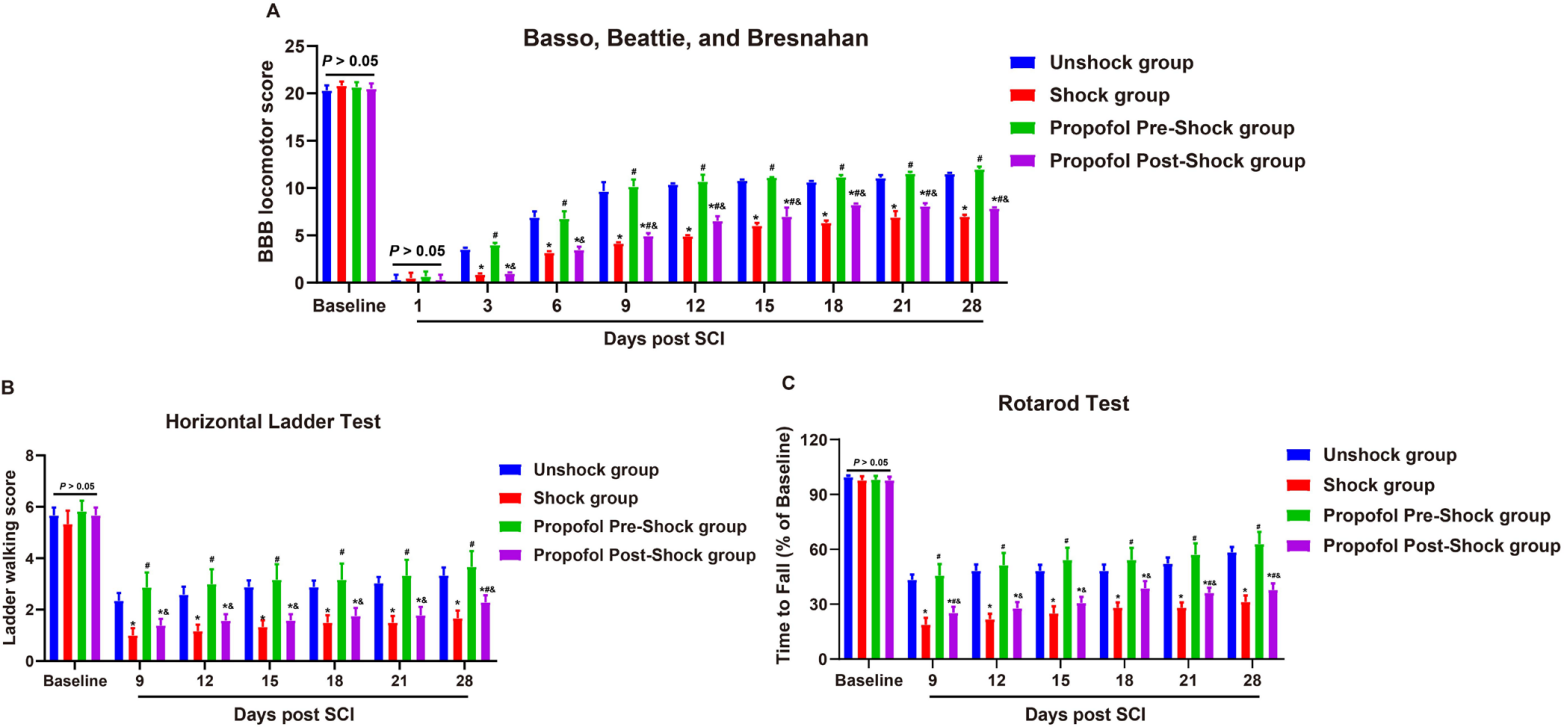
Effects of general anesthesia with preemptive propofol on functional recovery in SCI rats. (A) Basso, Beattie, and Bresnahan (BBB) locomotor rating scale scores assessed over 28 days. (B) Horizontal ladder test assessing hind‐limb placement accuracy. (C) Rotarod test measuring motor endurance and balance. Data are presented as mean ± SD. *p < 0.05 versus Unshock group; #*p <* 0.05 versus Shock group; &*p <* 0.05 versus Propofol Pre‐Shock group. *N* = 6.

The functional recovery of SCI rats was assessed using the BBB locomotor rating scale (Figure 6A), horizontal ladder test (Figure 6B), and rotarod test (Figure 6C), revealing consistent trends across measures. At baseline and day 1 post‐SCI, no significant differences were observed among groups (*p >* 0.05). From day 3 onward, distinct recovery patterns emerged. The BBB scores indicated that rats without pain stimulation exhibited the best locomotor recovery, while pain‐stimulated rats receiving propofol prior to stimulation significantly outperformed those with post‐stimulation administration (*p <* 0.05). Pain‐stimulated rats without intervention consistently showed the poorest recovery throughout the study period (*p <* 0.05). The Horizontal Ladder Test showed a similar trend, with significantly fewer foot misplacements in rats without pain stimulation (*p <* 0.05). Propofol administered prior to stimulation improved motor coordination compared to post‐stimulation administration (*p <* 0.05), while pain‐stimulated rats without intervention performed worst. The Rotarod Test, assessing motor endurance and balance, revealed that rats without pain stimulation maintained the highest percentage of baseline performance, while propofol administered prior to stimulation significantly improved outcomes compared to post‐stimulation administration (*p <* 0.05). Pain‐stimulated rats without intervention consistently exhibited the lowest performance. These findings underscore the critical role of early propofol intervention in improving locomotor recovery, motor coordination, and endurance following SCI.

## 4 Discussion

This study demonstrates the protective effects of general anesthesia with preemptive propofol administration in mitigating secondary damage and enhancing recovery in a SCI rat model. Specifically, preemptive intervention reduced pain‐induced hypertension, inflammation, and hemorrhage while preserving spinal cord integrity and promoting functional recovery. These findings highlight the multifaceted benefits of early propofol intervention under general anesthesia and suggest potential mechanisms underlying its neuroprotective effects.

Pain‐induced hypertension is a hallmark of secondary injury in SCI and is driven by autonomic dysregulation and persistent nociceptive input [24]. Our results indicated that pain stimulation triggered a significant hypertensive response, which was effectively mitigated by preemptive propofol administration. This reduction of SBP is likely mediated by the anesthetic's ability to suppress nociceptive signaling at supraspinal levels and its inhibitory effects on sympathetic outflow. Previous studies have shown that anesthetics such as pentobarbital and isoflurane prevent hypertension by modulating brainstem autonomic centers [4]. Propofol's known effects on gamma‐aminobutyric acid (GABA) receptor activation may also play a role in dampening the heightened sympathetic activity induced by pain stimulation [25, 26]. Importantly, post‐stimulation administration of propofol provided only transient suppression of SBP, underscoring the critical importance of timing in achieving sustained hemodynamic stability.

Hemorrhage is another major consequence of pain‐induced secondary injury in SCI, contributing to further vascular damage and exacerbating inflammation [27]. Our findings show that preemptive propofol administration significantly attenuated nociception‐induced increases in hemoglobin and alpha‐hemoglobin expression within the spinal cord. This protective effect may result from propofol's ability to stabilize endothelial integrity [28]. By preventing the breakdown of the BSCB, propofol likely limits the infiltration of blood components that amplify local inflammation and tissue damage [29]. In contrast, post‐stimulation intervention showed minimal efficacy, suggesting that early stabilization of the BSCB is critical in reducing hemorrhage‐associated injury.

The inflammatory response in SCI is a key driver of neuronal and glial damage [30]. Pain stimulation markedly elevated levels of IL‐1β, IL‐6, and TNF‐α in spinal cord tissue, reflecting an acute inflammatory surge. Preemptive administration of propofol significantly suppressed these inflammatory markers, likely through its ability to inhibit nuclear factor‐kappa B (NF‐κB) signaling and reduce the production of pro‐inflammatory cytokines [31]. This aligns with previous reports of propofol's anti‐inflammatory properties in various neurological conditions [13, 32]. Moreover, the attenuation of inflammation by propofol may also contribute to its effects on preserving vascular integrity and limiting secondary hemorrhage. Notably, post‐stimulation administration only partially reduced inflammatory cytokines, further emphasizing the importance of early intervention in breaking the cycle of inflammation and tissue damage.

Serum levels of GFAP and MBP provided additional insights into the neuroprotective effects of preemptive propofol administration. GFAP, a marker of astrocytic activation and gliosis, was significantly elevated following pain stimulation, indicating reactive gliosis in response to injury [33]. Preemptive propofol intervention reduced GFAP levels, suggesting mitigation of astrocytic overactivation and glial scar formation. This is important as excessive gliosis can impede axonal regeneration and exacerbate functional deficits. Similarly, MBP levels, indicative of myelin integrity, were better preserved in rats pretreated with propofol. By protecting oligodendrocytes from inflammatory damage, propofol helps maintain myelin integrity, which is essential for neural conductivity and recovery. Functional recovery observed in this study underscores the therapeutic potential of general anesthesia with preemptive propofol administration. Rats pretreated with propofol exhibited significant improvements in locomotor recovery, motor coordination, and balance across all behavioral assessments. The BBB locomotor rating scale revealed enhanced hind‐limb movement and coordination, likely reflecting reduced neuronal and vascular damage. Improved performance in the horizontal ladder test and rotarod test further demonstrated better sensorimotor integration and endurance. These functional benefits align with propofol's ability to reduce secondary injury mechanisms, including hypertension, inflammation, gliosis, and myelin loss, thereby creating a favorable environment for neural repair and plasticity.

While propofol exhibits neuroprotective effects, especially in mild ischemic conditions, its efficacy is limited compared to other anesthetics like nitrous oxide and fentanyl, which may offer more sustained protection in severe ischemic events [34]. Previous studies have shown that propofol reduces lipid peroxidation in SCI but does not enhance spinal cord structural integrity as effectively as thiopental [35]. Interestingly, intra‐aortic propofol injections have been shown to provide superior neuroprotective effects during thoracoabdominal aortic surgery, suggesting that the method of administration can influence its efficacy. These injections led to rapid recovery of motor‐evoked potential amplitudes, higher Tarlov scores, and better preservation of spinal cord motor neurons compared to intravenous propofol and saline controls [36]. Further studies in SCI models have demonstrated that propofol reduces excitotoxic spinal injury, attenuating cell death, particularly in motoneurons, through GABA(A) receptor‐mediated mechanisms [37]. This specificity to GABA(A) receptor activity may provide an advantage in clinical settings where targeted neuroprotection is crucial. In comparison to other anesthetics like etomidate, thiopental, and midazolam, propofol, along with midazolam, has been shown to offer superior neuroprotective effects in a fetal rat brain ischemia–reperfusion model [38], further supporting its robust neuroprotective properties. Moreover, propofol's neuroprotective effects are enhanced when combined with bone marrow stromal cells (BMSCs), leading to significant improvements in motor and electrophysiological function following SCI. This combination therapy outperformed the use of propofol or BMSCs alone, illustrating the potential of propofol to promote neurogenesis and recovery after SCI [39]. In clinical SCI management, propofol's neuroprotective effects, especially when combined with other therapeutic strategies, have strong translational relevance. Its ability to protect against excitotoxic injury, preserve spinal cord neurons, and improve functional recovery suggests its potential as a valuable adjunct to SCI treatment, particularly in high‐risk or traumatic contexts.

Although our study suggests that preemptive propofol may serve as a neuroprotective strategy in SCI, clinical SCI patients typically do not receive preemptive anesthesia unless undergoing planned surgeries. This presents a challenge for applying our findings directly in the emergency or acute care setting. The effectiveness of preemptive propofol may depend on the timing of intervention, as early administration is key to preventing secondary injury. In clinical practice, alternative anesthetic approaches, such as total intravenous anesthesia (TIVA) and multimodal analgesia, should be explored. These methods may offer similar neuroprotective effects while managing pain and stress in SCI patients. TIVA, for example, reduces reliance on volatile anesthetics and could provide a safer, more effective option for pain management in SCI patients [40]. While our findings are promising, further studies are needed to evaluate how preemptive anesthesia can be integrated into routine SCI care and to determine the optimal timing and combination with other anesthesia techniques.

Several limitations should be acknowledged that could affect the interpretation and generalizability of the results. Firstly, while it was hypothesized that propofol reduces pain‐induced hypertension through sympathetic modulation and nociceptive pathway regulation, the exact mechanism was not directly tested in this study due to the lack of autonomic and central nervous system measurements. This limits the understanding of propofol's precise action. Future studies should incorporate measurements of autonomic function and central nervous system activity to further validate these mechanisms. Secondly, the relatively small sample size raises concerns about statistical power, particularly when multiple comparisons (e.g., SBP, cytokines, and functional recovery assessments) were made. This limitation affects the reliability and generalizability of the results. Increasing the sample size and conducting post hoc power analysis would enhance the robustness of future findings. Lastly, although changes in GFAP and MBP levels provided insights into SCI and recovery, the lack of an SCI‐only control group makes it difficult to isolate the effects of pain stimulation from SCI itself. Including such a control group in future research would allow for a clearer assessment of propofol's neuroprotective effects. Additionally, the reliability of MBP as a biomarker in serum, particularly with a compromised blood–brain barrier, may limit the accuracy of assessing the treatment's effects. Alternative biomarkers, such as Neurofilament light chain (NfL) and Ubiquitin C‐terminal hydrolase L1 (UCH‐L1), could improve sensitivity in future studies.

## 5 Conclusion

Preemptive propofol administration under general anesthesia effectively mitigates pain‐induced hypertension, inflammation, and hemorrhage while preserving spinal cord integrity and enhancing functional recovery in SCI. These findings emphasize the importance of timely intervention and support the potential of general anesthetic strategies in SCI management. Further research is needed to optimize anesthesia techniques and explore their integration into clinical SCI care.

## Conflicts of Interest

The authors declare no conflicts of interest.

## Data Availability

The data that support the findings of this study are available from the corresponding author upon reasonable request.

## References

[1] A. Siglioccolo , R. Gammaldi , V. Vicinanza , et al., “Advance in Hyperbaric Oxygen Therapy in Spinal Cord Injury,” Chinese Journal of Traumatology 27, no. 6 (2024): 348–353. 37271686 10.1016/j.cjtee.2023.05.002 PMC11624306

[2] O. V. Alcantar‐Garibay , D. Incontri‐Abraham , and A. Ibarra , “Spinal Cord Injury‐Induced Cognitive Impairment: A Narrative Review,” Neural Regeneration Research 17, no. 12 (2022): 2649–2654. 35662196 10.4103/1673-5374.339475 PMC9165403

[3] R. C. Sterner and R. M. Sterner , “Immune Response Following Traumatic Spinal Cord Injury: Pathophysiology and Therapies,” Frontiers in Immunology 13 (2022): 1084101. 36685598 10.3389/fimmu.2022.1084101 PMC9853461

[4] J. A. Davis , A. C. Bopp , M. K. Henwood , P. Bean , and J. W. Grau , “General Anesthesia Blocks Pain‐Induced Hemorrhage and Locomotor Deficits After Spinal Cord Injury in Rats,” Journal of Neurotrauma 40, no. 23–24 (2023): 2552–2565. 36785968 10.1089/neu.2022.0449 PMC10698800

[5] M. E. Bakkali , S. Aboudrar , T. Dakka , and H. Benjelloun , “Autonomic Dysregulation and Phenobarbital in Patients With Masked Primary Hypertension,” Heliyon 6, no. 1 (2020): e03239. 32042972 10.1016/j.heliyon.2020.e03239 PMC7002820

[6] L. A. Dobrynina , K. V. Shamtieva , E. I. Kremneva , et al., “Daily Blood Pressure Profile and Blood‐Brain Barrier Permeability in Patients With Cerebral Small Vessel Disease,” Scientific Reports 12, no. 1 (2022): 7723. 35545641 10.1038/s41598-022-11172-1 PMC9095696

[7] M. M. Strain , M. A. Hook , J. D. Reynolds , Y. J. Huang , M. K. Henwood , and J. W. Grau , “A Brief Period of Moderate Noxious Stimulation Induces Hemorrhage and Impairs Locomotor Recovery After Spinal Cord Injury,” Physiology & Behavior 212 (2019): 112695. 31647990 10.1016/j.physbeh.2019.112695 PMC7326330

[8] A. Jurcau and A. Simion , “Neuroinflammation in Cerebral Ischemia and Ischemia/Reperfusion Injuries: From Pathophysiology to Therapeutic Strategies,” International Journal of Molecular Sciences 23, no. 1 (2021): 14, 10.3390/ijms23010014. 35008440 PMC8744548

[9] R. R. Ji , A. Nackley , Y. Huh , N. Terrando , and W. Maixner , “Neuroinflammation and Central Sensitization in Chronic and Widespread Pain,” Anesthesiology 129, no. 2 (2018): 343–366. 29462012 10.1097/ALN.0000000000002130 PMC6051899

[10] M. M. Strain , D. T. Johnston , R. E. Baine , et al., “Hemorrhage and Locomotor Deficits Induced by Pain Input After Spinal Cord Injury Are Partially Mediated by Changes in Hemodynamics,” Journal of Neurotrauma 38, no. 24 (2021): 3406–3430. 34652956 10.1089/neu.2021.0219 PMC8713547

[11] D. W. Hewson , N. M. Bedforth , and J. G. Hardman , “Spinal Cord Injury Arising in Anaesthesia Practice,” Anaesthesia 73, no. Suppl 1 (2018): 43–50. 29313911 10.1111/anae.14139

[12] H. Zheng , X. Xiao , Y. Han , et al., “Research Progress of Propofol in Alleviating Cerebral Ischemia/Reperfusion Injury,” Pharmacological Reports 76, no. 5 (2024): 962–980. 38954373 10.1007/s43440-024-00620-6

[13] C. Sun , D. Liu , S. Gao , M. Xiu , and Z. Zhang , “Propofol Ameliorates Spinal Cord Injury Process by Mediating miR‐672‐3p/TNIP2 Axis,” Biochemical Genetics 62, no. 6 (2024): 4914–4928. 38379038 10.1007/s10528-024-10718-4

[14] Council NR , Guide for the Care and Use of Laboratory Animals, Eighth ed. (National Academies Press, 2011), 246. 21595115

[15] T. Takasusuki , S. Yamaguchi , S. Hamaguchi , and T. L. Yaksh , “Effects of General Anesthetics on Substance P Release and c‐Fos Expression in the Spinal Dorsal Horn,” Anesthesiology 119, no. 2 (2013): 433–442. 23708866 10.1097/ALN.0b013e31829996b6 PMC3788114

[16] H. N. Alves , A. L. da Silva , I. A. Olsson , J. M. Orden , and L. M. Antunes , “Anesthesia With Intraperitoneal Propofol, Medetomidine, and Fentanyl in Rats,” Journal of the American Association for Laboratory Animal Science 49, no. 4 (2010): 454–459. 20819392 PMC2919186

[17] W. N. Arifin and W. M. Zahiruddin , “Sample Size Calculation in Animal Studies Using Resource Equation Approach,” Malaysian Journal of Medical Sciences 24, no. 5 (2017): 101–105. 10.21315/mjms2017.24.5.11 PMC5772820 29386977

[18] C. C. Serdar , M. Cihan , D. Yucel , and M. A. Serdar , “Sample Size, Power and Effect Size Revisited: Simplified and Practical Approaches in Pre‐Clinical, Clinical and Laboratory Studies,” Biochem Medicine (Zagreb) 31, no. 1 (2021): 10502. 10.11613/BM.2021.010502 PMC7745163 33380887

[19] K. K. Zatroch , C. G. Knight , J. N. Reimer , and D. S. Pang , “Refinement of Intraperitoneal Injection of Sodium Pentobarbital for Euthanasia in Laboratory Rats (*Rattus Norvegicus*),” BMC Veterinary Research 13, no. 1 (2017): 60. 28222732 10.1186/s12917-017-0982-y PMC5320784

[20] D. M. Basso , M. S. Beattie , and J. C. Bresnahan , “A Sensitive and Reliable Locomotor Rating Scale for Open Field Testing in Rats,” Journal of Neurotrauma 12, no. 1 (1995): 1–21. 7783230 10.1089/neu.1995.12.1

[21] S. Hashemizadeh , Z. Gharaylou , S. Hosseindoost , et al., “Long‐Term Administration of Bumetanide Improve Functional Recovery After Spinal Cord Injury in Rats,” Frontiers in Pharmacology 13 (2022): 932487. 36339604 10.3389/fphar.2022.932487 PMC9628211

[22] D. H. Lee , D. Cao , Y. Moon , et al., “Enhancement of Motor Functional Recovery in Thoracic Spinal Cord Injury: Voluntary Wheel Running Versus Forced Treadmill Exercise,” Neural Regeneration Research 20, no. 3 (2025): 836–844. 38886956 10.4103/NRR.NRR-D-23-01585 PMC11433897

[23] A. D. Gaudet , L. K. Fonken , M. T. Ayala , et al., “Spinal Cord Injury in Rats Dysregulates Diurnal Rhythms of Fecal Output and Liver Metabolic Indicators,” Journal of Neurotrauma 36, no. 12 (2019): 1923–1934. 30501584 10.1089/neu.2018.6101 PMC10027348

[24] E. Dzierzak , N. Mamun , J. Cohen , and J. Delgado‐Lebron , “Pain‐Induced Autonomic Dysreflexia Secondary to Spinal Cord Injury With Significant Improvement After Spinal Cord Stimulator Implantation,” Interventional Pain Medicine 2, no. 2 (2023): 100254. 39238671 10.1016/j.inpm.2023.100254 PMC11373029

[25] M. M. Sahinovic , M. Struys , and A. R. Absalom , “Clinical Pharmacokinetics and Pharmacodynamics of Propofol,” Clinical Pharmacokinetics 57, no. 12 (2018): 1539–1558. 30019172 10.1007/s40262-018-0672-3 PMC6267518

[26] V. Chidambaran , A. Costandi , and A. D'Mello , “Propofol: A Review of Its Role in Pediatric Anesthesia and Sedation,” CNS Drugs 29, no. 7 (2015): 543–563. 26290263 10.1007/s40263-015-0259-6 PMC4554966

[27] J. A. Davis , A. C. Bopp , M. K. Henwood , R. E. Baine , C. C. Cox , and J. W. Grau , “Pharmacological Transection of Brain‐Spinal Cord Communication Blocks Pain‐Induced Hemorrhage and Locomotor Deficits After Spinal Cord Injury in Rats,” Journal of Neurotrauma 37, no. 15 (2020): 1729–1739. 32368946 10.1089/neu.2019.6973 PMC7368389

[28] W. Chen , X. Z. Ju , Y. Lu , X. W. Ding , C. H. Miao , and J. W. Chen , “Propofol Improved Hypoxia‐Impaired Integrity of Blood‐Brain Barrier via Modulating the Expression and Phosphorylation of Zonula Occludens‐1,” CNS Neuroscience & Therapeutics 25, no. 6 (2019): 704–713. 30680941 10.1111/cns.13101 PMC6515893

[29] Y. Zhou , Y. Bai , P. Zhang , P. Weng , and W. Xie , “Propofol Alleviates Spinal Cord Ischemia‐Reperfusion Injury by Preserving PI3K/AKT/GIT1 Axis,” Journal of Investigative Medicine 72, no. 7 (2024): 705–714. 38715211 10.1177/10815589241254044

[30] M. B. Orr and J. C. Gensel , “Spinal Cord Injury Scarring and Inflammation: Therapies Targeting Glial and Inflammatory Responses,” Neurotherapeutics 15, no. 3 (2018): 541–553. 29717413 10.1007/s13311-018-0631-6 PMC6095779

[31] L. J. Xie , J. X. Huang , J. Yang , et al., “Propofol Protects Against Blood‐Spinal Cord Barrier Disruption Induced by Ischemia/Reperfusion Injury,” Neural Regeneration Research 12, no. 1 (2017): 125–132, 10.4103/1673-5374.199004. 28250758 PMC5319217

[32] S. Y. D. Leung , F. Meng , J. Liu , et al., “Sub‐Anaesthetic Dose of Propofol Attenuates Mechanical Allodynia in Chronic Post‐Ischaemic Pain via Regulation of PTEN/PI3K/IL‐6 Signalling,” Molecular Pain 19 (2023): 17448069231185232. 37314769 10.1177/17448069231185232 PMC10293517

[33] M. Cha , S. W. Um , M. Kwon , T. S. Nam , and B. H. Lee , “Repetitive Motor Cortex Stimulation Reinforces the Pain Modulation Circuits of Peripheral Neuropathic Pain,” Scientific Reports 7, no. 1 (2017): 7986. 28801619 10.1038/s41598-017-08208-2 PMC5554204

[34] Y. Kotani , M. Shimazawa , S. Yoshimura , T. Iwama , and H. Hara , “The Experimental and Clinical Pharmacology of Propofol, an Anesthetic Agent With Neuroprotective Properties,” CNS Neuroscience & Therapeutics 14, no. 2 (2008): 95–106. 18482023 10.1111/j.1527-3458.2008.00043.x PMC6494023

[35] E. Kaptanoglu , S. Sen , E. Beskonakli , et al., “Antioxidant Actions and Early Ultrastructural Findings of Thiopental and Propofol in Experimental Spinal Cord Injury,” Journal of Neurosurgical Anesthesiology 14, no. 2 (2002): 114–122. 11907391 10.1097/00008506-200204000-00005

[36] H. Kumagai , M. Isaka , Y. Sugawara , et al., “Intra‐Aortic Injection of Propofol Prevents Spinal Cord Injury During Aortic Surgery,” European Journal of Cardio‐Thoracic Surgery 29, no. 5 (2006): 714–719. 16522369 10.1016/j.ejcts.2006.01.042

[37] D. Bajrektarevic and A. Nistri , “Delayed Application of the Anesthetic Propofol Contrasts the Neurotoxic Effects of Kainate on Rat Organotypic Spinal Slice Cultures,” Neurotoxicology 54 (2016): 1–10. 26947011 10.1016/j.neuro.2016.03.001

[38] F. Harman , A. E. Hasturk , M. Yaman , et al., “Neuroprotective Effects of Propofol, Thiopental, Etomidate, and Midazolam in Fetal Rat Brain in Ischemia‐Reperfusion Model,” Child's Nervous System 28, no. 7 (2012): 1055–1062. 10.1007/s00381-012-1782-0 22562195

[39] Y. X. Wang , J. J. Sun , M. Zhang , et al., “Propofol Injection Combined With Bone Marrow Mesenchymal Stem Cell Transplantation Better Improves Electrophysiological Function in the Hindlimb of Rats With Spinal Cord Injury Than Monotherapy,” Neural Regeneration Research 10, no. 4 (2015): 636–643. 26170827 10.4103/1673-5374.155440 PMC4424759

[40] H. Asif , S. E. H. Tsan , A. Zoumprouli , M. C. Papadopoulos , and S. Saadoun , “Evolving Trends in the Surgical, Anaesthetic, and Intensive Care Management of Acute Spinal Cord Injuries in the UK,” European Spine Journal 33, no. 3 (2024): 1213–1222. 38217717 10.1007/s00586-023-08085-6

